# Supplementary material for: Cortex-wide BOLD fMRI activity reflects locally-recorded slow oscillation-associated calcium waves
Source: eLife. 2017 Sep 15;6:e27602. doi: 10.7554/eLife.27602 (PMC5658067; doi:10.7554/eLife.27602)
Supplement: Figure 3—source data 1. — Mean T-value over all ROIs for canonical HRF: 3.95 ± 0.53, mean T-value for FIR-extracted HRF: 3.91 ± 0.32. Means do not differ significantly between groups (Wilcoxon rank-sum test). [file elife-27602-fig3-data1.docx]

| *experimental HRF* | | *canonical HRF* | |  |
| --- | --- | --- | --- | --- |
|  | cluster size | T-value | cluster size | T-value |
| animal C1 E1 | 639 | 9.93 | 348 | 11.62 |
| animal C1 E2 | 351 | 12.41 | 1195 | 16.96 |
| animal C2 | 2172 | 14.30 | 1571 | 21.85 |
| animal C3 | 101 | 9.73 | 5 | 6.50 |
| animal C4 | 128 | 8.78 | 231 | 9.93 |
| animal C5 | 563 | 20.08 | 463 | 15.99 |
| animal T1 | 2561 | 20.63 | 1753 | 21.51 |
| animal T2 | 1750 | 14.47 | 1047 | 12.95 |

**Figure 3 Source Data 1**

**Figure 3 Source Data 1 - Table 1. Cluster sizes and T-values for experimental HRF versus canonical HRF for all experiments (Table for data shown in Figure 3 Supplement 2).** Mean T-value over all ROIs for canonical HRF: 3.95 ± 0.53, mean T-value for FIR-extracted HRF: 3.91 ± 0.32. Means do not differ significantly between groups (Wilcoxon rank-sum test).
