# Supplementary material for: Cortex-wide BOLD fMRI activity reflects locally-recorded slow oscillation-associated calcium waves
Source: eLife. 2017 Sep 15;6:e27602. doi: 10.7554/eLife.27602 (PMC5658067; doi:10.7554/eLife.27602)
Supplement: Figure 6—source data 1. — Number of clusters (>300 voxel) differ significantly between conditions (two-sample t-test (6)=−2.5538, p=0.0433). [file elife-27602-fig6-data1.docx]

|  | *slow wave activity ROI S1* | | *persistent activity ROI S1* | |  |
| --- | --- | --- | --- | --- | --- |
|  | Voxel  (cortex) | No. of clusters  (>300 voxel) | Correlation with pan-cortical IC (r) | Voxel  (cortex) | No. of clusters  (>300 voxel) |
| animal M7 | 76,237 | 1 | 0.80 | 15,751 | 6 |
| animal M8 | 13,908 | 1 | 0.55 | 8,400 | 4 |
| animal M9 | 19,950 | 2 | 0.49 | 12,649 | 2 |
| animal M10 | 47,650 | 3 | 0.64 | 7,863 | 5 |
|  | | | | | |
| mean | 39,436 | 1.75 | 0.62 | 11,165 | 4.25 |
| st dev | 24,764 | 0.83 | 0.12 | 3,231 | 1.48 |
| std error | 12,382 | 0.41 | 0.06 | 1,615 | 0.74 |

**Figure 6 Source Data 1**

**Figure 6 Source Data 1 - Table 2. Values for seed-based correlation between S1 and cortex during slow wave and persistent activity (n=4 animals; table for data shown in Figure 6 and Figure 6 Supplement 1).** Number of clusters (>300 voxel) differ significantly between conditions (two-sample t-test (6) = -2.5538, p = 0.0433).
