# Supplementary material for: Cortex-wide BOLD fMRI activity reflects locally-recorded slow oscillation-associated calcium waves
Source: eLife. 2017 Sep 15;6:e27602. doi: 10.7554/eLife.27602 (PMC5658067; doi:10.7554/eLife.27602)
Supplement: Figure 6—source data 2. — Number of clusters (>300 voxel) differ significantly between conditions(two-sample t-test (6)= −3.6667, p=0.0105). [file elife-27602-fig6-data2.docx]

|  | *slow wave activity ROI hf* | | *persistent activity ROI hf* | |  |
| --- | --- | --- | --- | --- | --- |
|  | Voxel  (cortex) | No. of clusters  (>300 voxel) | Correlation with pan-cortical IC (r) | Voxel  (cortex) | No. of clusters  (>300 voxel) |
| animal M7 | 142,760 | 4 | 0.70 | 16,644 | 12 |
| animal M8 | 19,945 | 3 | 0.25 | 16,820 | 9 |
| animal M9 | 23,105 | 6 | 0.53 | 22,395 | 11 |
| animal M10 | 32,576 | 9 | 0.67 | 10,570 | 12 |
|  | | | | | |
| mean | 54,597 | 5.5 | 0.54 | 16,607 | 11 |
| st dev | 51,113 | 2.29 | 0.18 | 4,183 | 1.22 |
| std error | 25,556 | 1.15 | 0.09 | 20,921 | 0.61 |

**Figure 6 Source Data 2**

**Figure 6 Source Data 2 - Table 3. Values for seed-based correlation between hippocampal formation (hf) and cortex during slow wave and persistent activity (n=4 animals; table for data shown in Figure 6 Supplement 2).** Number of clusters (>300 voxel) differ significantly between conditions(two-sample t-test (6) = -3.6667, p = 0.0105).
