# Supplementary material for: Cortex-wide BOLD fMRI activity reflects locally-recorded slow oscillation-associated calcium waves
Source: eLife. 2017 Sep 15;6:e27602. doi: 10.7554/eLife.27602 (PMC5658067; doi:10.7554/eLife.27602)
Supplement: Transparent reporting form [file elife-27602-transrepform.docx]

***eLife’s* transparent reporting form**

We encourage authors to provide detailed information *within their submission* to facilitate the interpretation and replication of experiments. If you have any questions, please contact us: [editorial@elifesciences.org](mailto:editorial@elifesciences.org).

**Sample-size estimation**

- You should state whether an appropriate sample size was computed when the study was being designed
- You should state the statistical method of sample size computation and any required assumptions
- If no explicit power analysis was used, you should describe how you decided what sample (replicate) size (number) to use

Please outline where this information can be found within the submission (e.g., page numbers or figure legends), or explain why this information doesn’t apply to your submission:

The objective of this study was to assess the relationship between slow calcium wave events and the fMRI BOLD signal.

The sample size (n=36 animals) is in line with previous work in the field. It is important to note, that we do not compare conditions, but rather asked whether there is a relation between slow wave onsets and the simultaneously acquired BOLD signal. The robustness of the studied phenomenon allowed us to observe clear effects between the two signals under investigation. The length and the stability of the recordings allowed detection of a solid relationship verified with the three analysis approaches employed: the model-based event-related analysis (based on a hemodynamic response model extracted from the raw data, as well as based on the canonical HRF model) and the additionally performed model-free seed-based correlation and independent component analysis.

These robust effects serve as a *post hoc* verification that the number of independent experiments was adequate for establishing the relation between slow wave onsets and fMRI. In total we tested the relationship between calcium and BOLD signals using almost 2000 slow calcium events (exact number of detected slow wave events: 1969) in a total of >2.8 h of recording time.

For the quantification of the temporal dynamics of slow calcium waves detected either with OGB-1 or GCaMP6f as calcium indicator, we considered 30 calcium events per group. This was based on the assumption of an estimated effect size *d* of 0.8, *power* (1 – β err prob) and α err prob of 0.05 which are considered adequate values for these parameters (yielding needed sample sizes of 27).

**Replicates**

- You should report how often each experiment was performed
- You should include a definition of biological versus technical replication
- The data obtained should be provided and sufficient information should be provided to indicate the number of independent biological and/or technical replicates
- If you encountered any outliers, you should describe how these were handled
- Criteria for exclusion/inclusion of data should be clearly stated
- High-throughput sequence data should be uploaded before submission, with a private link for reviewers provided (these are available from both GEO and ArrayExpress)

Please outline where this information can be found within the submission (e.g., page numbers or figure legends), or explain why this information doesn’t apply to your submission:

All of this information is provided in detail in the Material and Methods section.

**Statistical reporting**

- Statistical analysis methods should be described and justified
- Raw data should be presented in figures whenever informative to do so (typically when N per group is less than 10)
- For each experiment, you should identify the statistical tests used, exact values of N, definitions of center, methods of multiple test correction, and dispersion and precision measures (e.g., mean, median, SD, SEM, confidence intervals; and, for the major substantive results, a measure of effect size (e.g., Pearson's r, Cohen's d)
- Report exact p-values wherever possible alongside the summary statistics and 95% confidence intervals. These should be reported for all key questions and not only when the p-value is less than 0.05.

We describe the statistical procedures in detail in the Methods section and report means and SDs as well as exact p-values in the Results section (in some cases directly in the corresponding figure legends).

Excerpts of recorded calcium traces are shown next to the BOLD data of each experiment (Figure 3A and Figure3 – figure supplement1 A) and for the slow wave detection method in Figure 2 A and B. Figure 1 G and H shows additional calcium traces, Figure 1 M shows calcium and LFP traces.

Please outline where this information can be found within the submission (e.g., page numbers or figure legends), or explain why this information doesn’t apply to your submission:

(For large datasets, or papers with a very large number of statistical tests, you may upload a single table file with tests, Ns, etc., with reference to page numbers in the manuscript.)

**Additional data files (“source data”)**

- We encourage you to upload relevant additional data files, such as numerical data that are represented as a graph in a figure, or as a summary table
- Where provided, these should be in the most useful format, and they can be uploaded as “Source data” files linked to a main figure or table
- Include model definition files including the full list of parameters used
- Include code used for data analysis (e.g., R, MatLab)
- Avoid stating that data files are “available upon request”

Please indicate the figures or tables for which source data files have been provided:

We provide the spm batch files containing the model specifications (Source Code file) as well as the vectors containing the slow wave onsets. The slow wave detection algorithm was already published by Seamari et al. in 2007 and we used only minor adaptations for the application of this algorithm to optic-fiber calcium data, described in detail in the method section. The correlation and independent component analyses were performed as described in the Methods section using standard procedures or freely available toolboxes (e.g. http://research.ics.aalto.fi/ica/fastica/). Numerical data of quantifications shown in Figure 1 I,J,K,L are provided as Excel files.
